# Production of monoclonal antibody of heat-labile toxin A subunit to identify enterotoxigenic *Escherichia coli* by epitope mapping using synthetic peptides

**DOI:** 10.3389/fimmu.2023.1152910

**Published:** 2023-05-18

**Authors:** Jun-Young Park, Seung-Hak Cho

**Affiliations:** ^1^ Division of Zoonotic and Vector Borne Disease Research, Center for Infectious Disease Research, Korea National Institute of Health, Cheongju, Republic of Korea; ^2^ Environmental Diseases Research Center, Korea Research Institute of Bioscience and Biotechnology, Daejeon, Republic of Korea

**Keywords:** enterotoxigenic *Escherichia coli* (ETEC), heat-labile toxin (LT), epitope mapping, peptide, monoclonal antibody

## Abstract

**Background:**

Enterotoxigenic *Escherichia coli* (ETEC) is a major cause of diarrhea through two enterotoxins, a heat-labile toxin and a heat-stable toxin. These toxins alter the cellular signaling pathways, ultimately triggering an increase in chloride secretion and watery diarrhea.

**Objective:**

For the development of an ETEC vaccine, we attempted to construct a peptide-specific monoclonal antibody library against heat-labile enterotoxin A subunit (LT-A) by epitope mapping using synthetic peptides.

**Methods:**

Sera produced by five mice immunized with recombinant LT-A protein were examined for specific recognition with synthetic 15-mer and 34-mer peptides of LT-A proteins using enzyme-linked immunosorbent assay. The analysis revealed that the synthetic peptides number 8, 16, 24, 33, 36, 38, and 39 reacted with an anti-LT-A polyclonal antibody. For the possible prediction of LT-A epitopes, each full-length protein sequence was subjected to BCPreds analysis and three-dimensional protein structure analysis. The data showed that three peptides (synthetic peptide numbers: 33, 36, and 38–39) have identical antigenic specificities with LT-A protein, suggesting the usefulness of these linear peptide epitopes.

**Results:**

Based on these peptides, we produced monoclonal antibodies to improve the specificity of LT-A detection. Monoclonal antibodies produced from two peptides (numbers 33 and 36) showed affinity for an LT-A recombinant antigen. Moreover, peptide epitope prediction analysis showed that the sites of the three peptides were identical to those exhibiting actual antigenicity. Also, it was confirmed that the amino acid sequence that actually showed antigenicity was included in the peptide predicted only by ETEC-LT-A-33. Also, the specificity of the antibody for ETEC-LT-A-33 was validated using bacterial cells, and the neutralizing effect of the antibody was determined by assessing cytokine release in infected HCT-8 cells.

**Conclusion:**

The monoclonal antibodies produced in this study are useful toolsfor vaccine production against ETEC and can be used to identify peptide antigencandidates.

## Introduction

Enterotoxigenic *Escherichia coli* (ETEC) causes diarrhea and diarrheal death among young children and travelers in developing countries. The infection caused by this bacterium is responsible for fatalities, with an estimated 800,000 deaths each year due to diarrhea, making it the second leading cause of death among children under the age of five in 2019, and at least 370,000 children dying from this disease in the same year (1–3). ETEC colonizes the mucosal surface of small intestines and causes severe diarrhea, dysentery, abdominal cramps, and fever. This infection can be life threatening due to substantial fluid loss and severe dehydration (4, 5). The major virulence factors of diarrhea-causing ETEC strains are enterotoxins, specifically a heat-labile toxin (LT) and a heat-stable toxin. These toxins alter the cellular signaling pathways, ultimately triggering an increase in chloride secretion and watery diarrhea (6, 7). The LT is an AB5 toxin encoded by the *elt*AB operon with similarities to cholera toxin; it binds to ADP and ribosylates the guanyl-nucleotide alpha regulatory binding protein of the adenylate cyclase system, thereby increasing the levels of cyclic AMP (8, 9). Recently, it has been reported that the A subunit of LT (LT-A) possesses adjuvant properties in addition to toxicity (10, 11).

Epitope mapping has become increasingly important in both vaccine and antibody drug development (12, 13). Knowledge of the epitopes of an antibody will markedly facilitate drug design, vaccine development vaccines, and diagnosis. Notably, synthetic peptides are increasingly replacing biological molecules in diagnostic tests (14, 15). Moreover, unequivocally characterized peptide antigens can display enhanced specificity for recognition, thereby eliminating or minimizing potential cross-reactivity between structurally homologous protein epitopes (16–18). Individual antigenic epitope mapping of native proteins provides useful information that can assist in the design of peptide-based diagnostic tests and peptide libraries for monitoring specific cellular immunity in patients (19–21). We have attempted to construct a peptide-specific monoclonal antibody library against LT-A by epitope mapping utilizing synthetic peptides for the production of an ETEC vaccine.

The currently available ETEC vaccine is a recombinant vaccine that combines four recombinant E. coli strains that overproduce adhesins CFA/I, CS3, CS5, and CS6 with a recombinant protein, LCTBA (CTB/LTB hybrid B subunit protein), which is a hybrid B subunit of heat-labile enterotoxin and cholera toxin (22). This recombinant vaccine seemed to be safe and well tolerated in children aged 6–59 months when administered orally, as evidenced by the absence of treatment-related serious adverse events (23). The basis for this approach was that a higher immunogenic response may arise from the binding of a weak immunogen to the carrier molecule. Recombinant molecules can also be used in this process. Moreover, recombinant technology offers a rapid and cost-effective method for the creation of toxoids compared with coupling processes.

The main goal of the current research effort focusing on the development of an ETEC vaccine was to induce immune responses against colonization factors (CFs) and one or both ETEC toxins, which contribute to protective immunity (24, 25). In addition, several of the existing candidate CF/CS antigens may cross-react with antibodies, potentially expanding coverage (26, 27). CF/coli surface (CF/CS) antigens vary considerably; thus, vaccines must express epidemiologically frequent CF/CS antigens and toxin components. A vaccine produced using four-to-five such components would protect against 70%–80% of the most prevalent strains linked to illness.

While cross-reactivity has been exploited to develop a recombinant vaccine for LT-A, the design of peptides for an ETEC vaccine has not been reported, and the effectiveness of current vaccination strategies in preventing enteric infections is still unclear. Therefore, the current study aimed to develop a more specific approach for the prevention and treatment of ETEC infection, a major cause of diarrhea, by identifying optimal vaccine candidates. Herein, for the development of a vaccine against ETEC, we used an epitope-mapping strategy to select synthetic antigenic probes that can recognize specific antibodies against LT-A of ETEC through an enzyme-linked immunosorbent assay (ELISA). Peptide-specific antibodies can be easily produced and, thus, are very useful. To this end, a library of overlapping peptides was synthesized and tested using ELISA, to characterize the linear putative antigenic epitopes of LT-A. Moreover, we produced a monoclonal antibody that can be considered a useful tool and was used to identify a peptide antigen candidate.

## Materials and methods

### Construction and expression of recombinant protein

**Figure 1:**
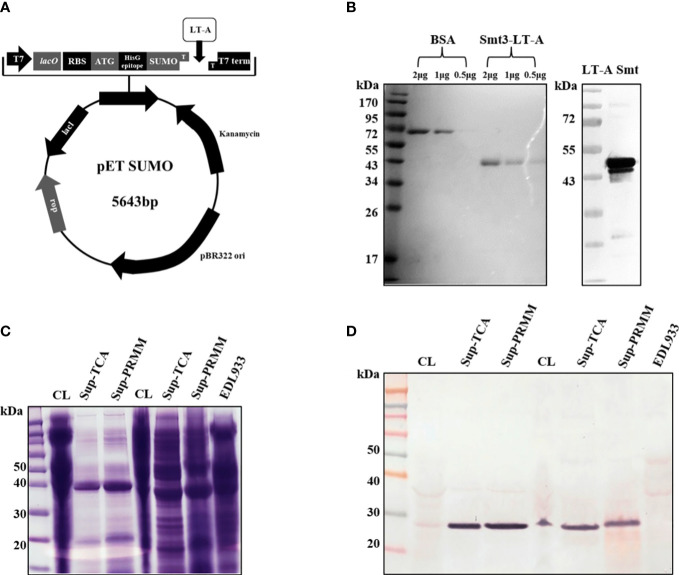
Schematic of SUMO-LT-A expression vector and detection of LT-A subunit by anti-LT-A antibody. **(A)** Schematic of SUMO-LT-A expression vector. The coding sequence of LT-A was cloned into pET SUMO vector. pBR322 ori, replication origin; Kanamycin, coding sequence of kanamycin resistance gene; SUMO, coding sequence of small ubiquitin-like modifier (SUMO); HisG epitope, 6× His tag coding sequence; ATG, start codon; RBS, ribosome binding site; lacO, lac operator; T7, T7 promoter; **(B)** Heat-labile toxin A subunit purification using an Ni-NTA column and an ion exchange (Q) column and detection of the LT-A subunit by the anti-LT-A antibody. **(C)** Stained Bis-Tris acrylamide gel with Coomassie blue solution. **(D)** Purification of the toxin protein from bacterial cells and supernatant. PVDF membrane immunostained with the anti-LT-A antibody.

ETEC *Escherichia coli* strain H10407 (O78:H11) was used to isolate the LT-A gene. This strain was cultured in Luria–Bertani (LB) medium. The gene fragment encoding LT-A was amplified through polymerase chain reaction using ETEC H10407 genomic DNA with designed primers (**Tables 1**, **2**). Subsequently, it was cloned into a pETSUMO vector (Novagen, Darmstadt, Germany) through ligase-independent cloning methods (**Figure 1A**). LT-A was expressed in *Escherichia coli* BL21 (DE3) cells. Cells were grown to an optical density (at 600 nm) of 0.6 in LB broth containing kanamycin at 37°C. The expression of recombinant LT-A protein was induced with 0.1 mM isopropyl-D-1-thiogalactopyranoside (IPTG). At 4 h after induction, cells were harvested by centrifugation at 5,000×*g* for 30 min. Cell pellets were suspended in lysis buffer containing 50 mM NaH_2_PO_4_/Na_2_HPO_4_ (pH 7.4), 500 mM NaCl, and ethylenediaminetetraacetic acid-free protease inhibitor cocktail (Roche, Indianapolis, IN, USA). The suspension was incubated at room temperature with lysozyme for 1 h, followed by cell lysis through sonication. Cell debris was removed by centrifugation at 40,000×*g*. The supernatant was loaded onto a His column (GE Healthcare, Little Chalfont, UK) pre-equilibrated with buffer A (50 mM NaH_2_PO_4_/Na_2_HPO_4_ [pH 7.4], 50 mM imidazole, 500 mM NaCl). The column was washed with buffer A, and proteins were eluted with buffer B (50 mM NaH_2_PO_4_/Na_2_HPO_4_ [pH 7.4], 500 mM NaCl, 50–300 mM imidazole in a linear gradient). Fractions containing the protein were pooled and dialyzed using buffer C (20 mM Tris-HCl [pH 7.4], 50 mM NaCl, 1 mM ethylenediaminetetraacetic acid). The purified preparations were stored at −70°C until use.

**Table 1:** *Escherichia coli* strain used in this study.

Strain	Relevant properties	Source of reference
BL21	B F^-^ *ompT hsdS(r*_B_ *^-^ m*_B_ *^-^) gal dcm*	GE Healthcare
H10407	ETEC strain (LT^+^ STa^+^)	ATCC 35401

**Table 2:** LT-LIC primers used in this study.

Primer Name	Primer Sequence	Tm (full)	Tm (gene)
LT-LIC-F	AGATTGGTGGCAATGGCGACAAATTATACCGTG	70.4	53.4
LT-LIC-R	GAGGAGAGTTTAGACTCATAATTCATTCCGAATTCTGTTA	65.6	51.9

### Western blotting analysis

Purified recombinant toxin proteins were boiled in SDS-PAGE sample buffer and loaded in a 10% Bis-Tris acrylamide gel for 1 h. The separated protein bands were transferred onto a polyvinylidene difluoride (PVDF) membrane (Millipore, Darmstadt, Germany). The membrane was blocked with 5% blocking solution at 37°C for 1 h. Thereafter, the membrane was probed with the primary mouse anti-LT-A antibody (1:500) at 4°C overnight. The membrane was washed thrice for 10 min and incubated with alkaline phosphatase-conjugated anti-mouse IgG antibody (1:2,000; Sigma, St. Louis, MO, USA) at 37°C for 1 h. Protein bands were visualized using the 5-bromo4-chloro-3-indolyl phosphate/nitro blue tetrazolium liquid substrate system (Sigma, St. Louis, USA).

### Peptides synthesis

Several 15-mer and 34-mer peptides were synthesized using the Automated Multiple Peptide Synthesizers (GLbiochem, Shanghai, China). Subsequently, the purity of these peptides was evaluated through high-performance liquid chromatography (GLbiochem) and MS (GLbiochem). The synthesized peptides are shown in **Supplementary Table S1**.

### Peptide prediction and three-dimensional structure simulation

For the prediction of LT-A epitopes, each full-length protein sequence was subjected to BCPreds analysis (Imtech, Chandigarh, India), and predicted LT-A epitopes (15 mers and 33 mers) with a BCPreds cut-off value >0.8 were selected (http://tools.iedb.org/main/bcell/). The cut-off score >0.8 was preferred to get peptides resemble to maximum epitope-like properties (28). The DiscoTope Server was used to predict discontinuous LT-A epitopes from 3D protein structures (DiscoTope 2.0; DTU Health Tech, Lyngby, Denmark). Prediction of conformational LT-A epitopes from primary sequencing was performed using the web server CBTOPE (Imtech). Surface-exposed LT-A epitope sequences with a cut-off value for BCPreds >0.8 were selected and further analyzed using VaxiJen (http://www.jenner.ac.uk/VaxiJen) (29) (threshold = 0.4, ACC output) to determine antigenicity.

### Polyclonal antibody production

Adult female BALB/C mice (age: 6 weeks) were intraperitoneally immunized with 200 µL of Freud’s adjuvant (Sigma, St. Louis, USA) containing 50 µg of purified recombinant LT-A proteins. The mice received four injections with a 2-week interval between administrations. Serum was collected by cardiac puncture and stored at −80°C until use.

### Monoclonal antibody production

Female BALB/c mice (age: 4 weeks) were intraperitoneally injected with 100 µg of peptide emulsified in complete Freud’s adjuvant (Sigma). These mice were also each injected with 100 µg of peptide emulsified in incomplete Freud’s adjuvant (Sigma, St. Louis, USA), with a 2-week interval between administrations. The final intraperitoneal injection with saline was administered 4 days before the fusion. Fusions were performed using conventional methodology (30). Briefly, spleen cells (1 × 10^8^) obtained from immunized rabbits and the fusion partner Sp2/0 were fused at a ratio of 5:1 with 50% polyethylene glycol 1500 (Roche) at 37°C in serum-free medium. The cells were seeded in 96-well microtiter plates (~2 × 10^5^ spleen cells per well) in hypoxanthine-aminopterin-thymidine medium supplemented with 15% fetal bovine serum. Supernatants were tested for the presence of antibodies specific for the immunogen using ELISA. Hybridomas were cloned by limiting dilution in 96-well microtiter plates.

### ELISA

ELISA plates (Corning, NY, USA) were coated with 100 ng/100 µL of synthetic peptides at 4°C, overnight. Subsequently, the plates were washed thrice with phosphate-buffered saline containing 5% skim milk for 1 h at 37°C. Sera samples obtained from immunized mice were diluted (1:1,000 dilution) and dispensed into each well. After 2 h of incubation, horseradish peroxidase-conjugated goat anti-mouse IgG (AbClon, Seoul, Korea) (1:10,000 dilution) was added. After 1 h, color was performed with 3,3’,5,5’-tetramethylbenzidine solution for 15 min. The reaction was terminated by the addition of 1 N sulfuric acid solution, and the optical density was determined at 450 nm using the Victor X3 (PerkinElmer, Waltham, MA, USA) ELISA reader (31).

### Cell culture and measurement of pro-inflammatory cytokines

Human ileocecal adenocarcinoma (HCT-8) cells were obtained from the American Type Culture Collection (Manassas, VA, USA). These cells were cultured in Dulbecco’s modified Eagle’s medium supplemented with 10% fetal bovine serum, 4 mmol/L L-glutamine, 100 U/mL penicillin, and 100 U/mL streptomycin. The cells were maintained in a humidified incubator at 37°C with 5% CO_2_. The levels of pro-inflammatory cytokines, such as IL-8 and INF-γ in culture media were measured using an ELISA kit (Affymetrix, eBioscience) and cytokine-specific antibodies according to the instructions provided by the manufacturer.

### Statistical analysis

All experiments in this study were performed in triplicate. The data were analyzed using a one-way analysis of variance, followed by the *post hoc* Bonferroni test. The p-values <0.05 and <0.01 denoted statistically significant differences.

## Results

### Detection of LT-A protein by anti-LT-A polyclonal antibody

To evaluate the activity of anti-LT-A antibodies, we tested their ability for the detection of recombinant and endogenous LT-A protein. For this purpose, we constructed and expressed a recombinant LT-A protein (**Figure 1A**). The expression of LT-A by the recombinant pETSUMO-LT-A was tested using different concentrations of IPTG (0.1–1 mM) and protein induction times (4–12 h). The maximal expression of LT-A was detected under the following conditions: 0.1 mM IPTG and induction for 4 h at 37°C. Next, we purified LT-A protein using a nickel-nitriloacetic acid column. The lysates were subjected to 10% SDS-PAGE and stained with Coomassie brilliant blue. BL21:pETSUMO-LT-A was grown in basal LB medium. Cell lysates were subjected to SDS-PAGE. Western blotting analysis using an anti-toxin antibody revealed the corresponding LT-A recombinant protein at 43 kDa (**Figure 1B**). Bacterial culture supernatant and toxin protein in bacterial cells were identified to obtain crude enterotoxin of ETEC. The bacterial culture supernatant was precipitated with trichloroacetic acid and pyrogallol red-molybdate-methanol, to confirm the toxin protein (**Figures 1C, D**). Finally, the toxin protein was purified from bacterial culture supernatants using the pyrogallol red-molybdate-methanol precipitation method.

### Synthesis of 39 peptides through peptide overlapping and detection of the peptides by an anti-LT-A polyclonal antibody

**Figure 2:**
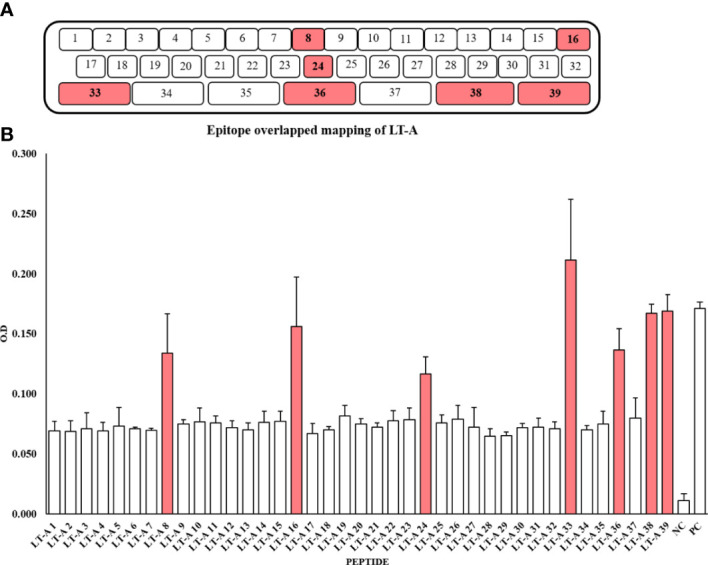
Synthesis of 39 peptides through peptide overlapping and detection of the peptides by an anti-LT-A polyclonal antibody. **(A)** Amino acids used in the production of peptides. **(B)** Detection of the 39 peptides by an anti-LT-A polyclonal antibody based on the ELISA results. The positive control (PC) consisted of the entire peptide, while a non-coated well served as the blank control, and the peptide library was incubated with the secondary antibody only to serve as the negative control (NC). The values shown in the figure represent the means ± standard deviation (n = 3).

For peptide synthesis, we considered peptide fragment length and degree of overlap (32, 33). There were 32 and seven 15-mer and 34-mer peptide fragments. The peptides overlapped three times (**Figure 2A**, **Supplementary Table S1**). For epitope mapping, serums produced by five mice immunized with the full-length recombinant LT-A protein were examined for the presence of antibodies specific to the synthetic peptides of LT-A by ELISA. It is expressed as the average O.D value of each ELISA assay (**Figure 2B**, **Supplementary Table S2**). As a result, the synthetic peptide numbers 8, 16, 24, 33, 36, 38 and 39 were detected. Those detected peptides are shaded in red in (**Figures 2A, B**).

### Monoclonal antibody production and antibody titer measurement using LT-A peptides 33, 36, and 38–39

**Figure 3:**
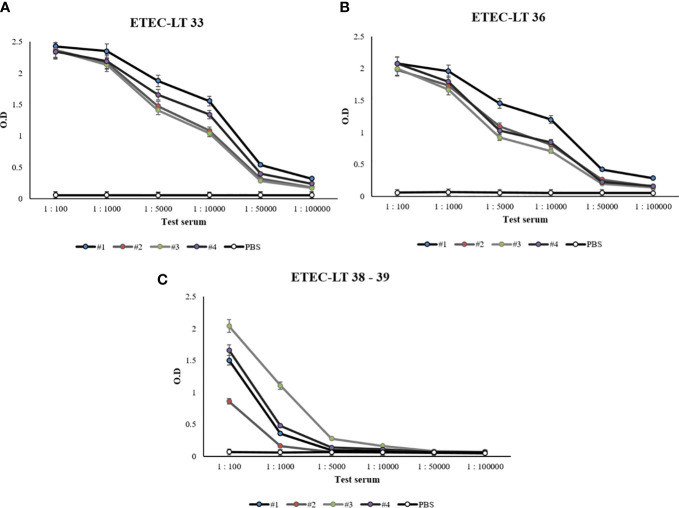
Anti-LT-A antibody titer measurement by ELISA. **(A)** ELISA data of anti-LT-A peptide 33, **(B)** ELISA data of anti-LT-A peptide 36 **(C)** ELISA data of anti-LT-A peptides 38–39. PBS was used as the negative control. The data are presented as mean ± SD of four mice per group at various dilution points.

The selected ETEC-LT-A-33, 36, 38–39 peptides were each injected into four mice. Prior to injection, to prevent the intracellular destruction of small peptides, BSA was conjugated to each of the three peptides. The reaction of the generated antibody with the synthesized peptide was analyzed using ELISA. The antibodies obtained through the immunization of mice with each of these peptides exhibited slightly different titers. Antibody titers of 1:10,000 were recorded for the ETEC-LT-A-33 and 36 peptides, whereas antibodies against the ETEC-LT-A-38–39 peptide were not generated (**Figures 3A–C**). Therefore, it was confirmed that the ETEC-LT-A-33 and -36 peptides were suitable for antibody production.

### Prediction of hydrophilicity analysis of the selected ETEC-LT-A peptides

**Figure 4:**
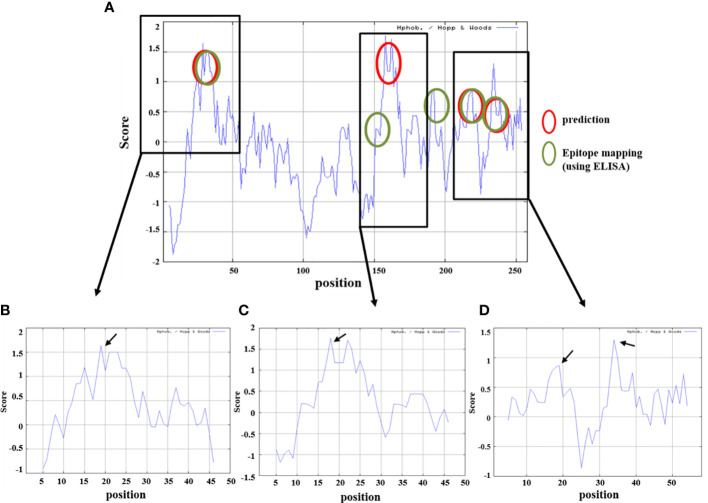
Prediction of hydrophilicity analysis of the selected ETEC-LT-A peptides. **(A)** Comparison of peptide prediction and amino acid sequences showing antigenicity. **(B)** Hydrophilicity of ETEC-LT-A-33, **(C)** hydrophilicity of ETEC-LT-A-36 and **(D)** hydrophilicity of ETEC-LT-A-38-39. X-axis: position; Y-axis: score; red circle: predicted region; and green circle: mapping region using ELISA.

**Figure 5:**
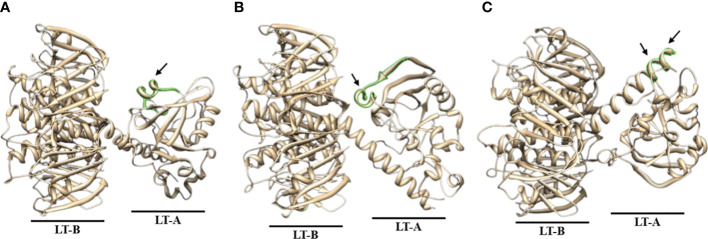
Predicted 3D structure analysis of ETEC-LT-A amino acids. **(A)** 3D structure of ETEC-LT-A-33. **(B)** 3D structure of ETEC-LT-A-36. **(C)** 3D structure of ETEC-LT-A-38-39. In each LT-A structure, the region highlighted in green represents the predicted protruding portion, while the predicted sequence segment is indicated by the arrow. The sequence for each structure is listed in **Supplementary Table S3**.

Antibody titer measurement through ELISA was performed to find a part that matches the amino acid sequence that actually showed antigenicity and the peptide prediction result through a program that predicts protein antigenicity, and to confirm the significance. The anti-LT-A polyclonal antibody binding was consistent with the predicted hydrophilicity of peptides. Hydrophilic properties are required for strong antigenicity (34). In **Figures 4B–D**, amino acids showing strong hydrophilicity in each part of the graph are indicated by black arrows. As a result, it was predicted that the ETEC-LT-A-33, 36, and 38–39 peptides were hydrophilic and may stimulate antibody production. The structure of the domain, including three types of hydrophilic peptides, was analyzed to evaluate whether the antigenic part is exposed to the outside because the interaction between the antigen and the antibody is an important factor for antibody production. It was confirmed that all amino acid regions, including the selected three peptides ETEC-LTA-33, 36, and 38–39, were exposed to the outside of the structure (**Figures 5A–C**).

For the prediction of LT-A epitopes, each full-length protein sequence was subjected to BCPreds analysis (Imtech) (35, 36). The data showed that the three peptides had specificity for the B-cell epitopes in LT-A. We subsequently checked whether this finding was consistent with the results of amino acid sequence and the experimental data that actually showed antigenicity. As shown in **Figures 4A–D**, the data from the peptide prediction analysis showed that the sites of the three peptides were identical to those showing actual antigenicity.

### Prediction of 3D structural analysis of the final selected ETEC-LT-A peptides

As shown in the figure below, it was confirmed that all amino acid regions, including the three selected peptides ETEC-LT-A-33 (RPPDEIKRSGGLMP-C), 36 (RLHRNREYRDRYYR-C), and 38–39 (C-RTITGDTCNEET, C-LRKYQSKVKRQI), were exposed to the outside of the structure (**Figures 5A–C**). It was confirmed that the amino acid sequence that actually showed antigenicity was included in the peptide predicted only by ETEC-LT-A-33 (**Supplementary Table S3**). Therefore, the ETEC-LT-A-33 peptide was selected for further analysis.

### ETEC-LT-A-33 and confirmation of antibody effects by measuring cytokine secretion in infected HCT-8 cells

**Figure 6:**
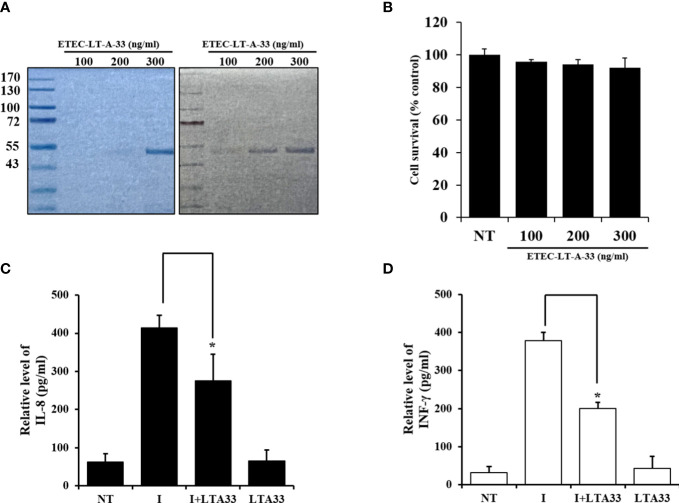
Production of cytokine by infected HCT-8 cells was suppressed by ETEC-LT-A-33. **(A)** Stained Bis-Tris acrylamide gel with Coomassie brilliant blue and immuno-stained PVDF membrane with ETEC-LT-A-33. **(B)** Cell viability measured by MTT assay. **(C)**, **(D)** Cytokines levels of IL-8 and INF-γ in HCT-8 infected cells. NT, none treatment; I, infection; LT-A-33: ETEC-LT-A-33. The data shown are presented as mean ± SD from independent experiments executed in triplicate. *p < 0.05 relative to HCT-8 infected cells.

Western blotting was used to determine whether ETEC-LT-A-33 could detect the ETEC-LT-A recombinant protein. The cells were treated with 100, 200, and 300 ng of ETEC-LT-A-33. The results of this analysis confirmed that ETEC-LT-A-33 detected the ETEC-LT-A recombinant protein (**Figure 6A**). Using the MTT (3-(4,5-dimethylthiazolyl-2)-2,5-diphenyltetrazolium bromide) assay, we assessed the proliferation of HCT-8 cells to investigate the effect of ETEC-LT-A-33 on cell viability. Treatment with 0–300 ng/mL ETEC-LT-A-33 did not result in cell death (**Figure 6B**). Thus, we used 0–300 ng/mL ETEC-LT-A-33 for all subsequent experiments. Moreover, after the challenge of infection in intestinal epithelial cells HCT-8, measurement of cytokine release was performed by ELISA to determine the antibody anti-inflammatory effect of the ETEC-LT-A-33 peptide. The results revealed that the levels of major inflammatory factors IL-8 and INF-γ (which are secreted after infection) were decreased (**Figures 6C, D**). These data confirmed that the antibody exerted a protective effect on HCT-8 intestinal cells.

## Discussion

Two enterotoxins produced by ETEC, a LT and a heat-stable toxin (ST), are a prominent causes of diarrhea (37, 38). We have attempted to construct a peptide-specific monoclonal antibody library against LT-A by epitope mapping utilizing synthetic peptides for the production of an ETEC vaccine.

Overlapping peptide libraries can be used for epitope mapping of selected protein portions and antigenic fragments that contribute to the immunological activity. Overlapping peptide libraries are ideal for the discovery of T-cell epitopes because the latter are short linear peptides derived from the primary sequence of the protein. These libraries are also appropriate for scanning the primary sequence of proteins for linear or ‘continuous’ B-cell epitopes (antibody-defined) (39–44). In this study, we synthesized a library of 39 peptides overlapping the N-terminal for the development of novel peptide-based diagnostic tools for ETEC. The discovery of a linear epitope in a region described as part of a large conformational epitope is surprising. Nevertheless, cross-reactivity of antibodies between peptides and folded proteins is possible. Examples of antibodies binding to short linear peptides that are part of large conformational epitopes have been previously reported (45, 46). Epitope mapping using synthetic overlapping peptides offers convenience, scalability, and reproducibility for the development of innovative diagnostic/prognostic tools, aiming to support the early clinical diagnosis of disease caused by infection with ETEC. Additional studies are warranted to investigate the importance of the selected peptides in this setting.

The main current vaccine candidates seem to be compatible with the general requirements of the most recent World Health Organization Preferred Product Characteristics for an acceptable ETEC vaccine (47). However, the effectiveness of existing vaccination strategies in protecting against enteric infections remains unknown. To date, the successful immunization of children against these illnesses using either the oral or parenteral route has been challenging. However, the double-mutant heat-labile toxin (dmLT) enhanced mucosal IgA responses as an adjuvant in children and infants; hence, it could assist in overcoming this obstacle. For instance, recent research on Bangladeshi infants aged 6–11 months demonstrated that dmLT enhanced the mucosal immune response to ETEC antigens after vaccination with ETVAX (48). Moreover, the inclusion of dmLT in the vaccine accelerated the kinetics of the immune response. Similar to dmLT, a mucosal adjuvant may help with dose-sparing, as well as enhance the candidates’ cost of goods and the Full Value of Vaccines Assessment (FVVA) (49). In the development of ETEC vaccines, cost effectiveness, appropriate distribution, safety/tolerance, and high immunogenicity should be taken into consideration for successful use in pediatric populations in low- and middle-income countries (50).

Here, we employed a more specific approach through the production of peptides, to develop useful and effective vaccines. This is the first investigation to demonstrate that synthetic peptide fragments of LT-A in ETEC are recognized as linear epitopes. Unlike ETEC vaccines using complexes, we can specify the target site using peptides, thereby increasing the efficiency of the vaccine.

The present results support the significance of conformational features in the antigen-antibody interaction that are extensively reported in the literature. Our findings provide important insights into the nature of the LT-A-antibody interaction that characterizes protein epitopes. Synthetic peptides number 8, 16, 24, 33, 36, 38, and 39 reacted with the polyclonal anti-LT-A antibody. Each full-length protein sequence was subjected to BCPreds analysis and 3D protein structure analysis for 15mer short peptide prediction of LT-A comprising B-cell epitopes. The data revealed that three other peptides (synthetic peptide numbers 33, 36, and 38–39) have the same antigenic specificities as the LT-A protein, suggesting the potential usefulness of epitopes. We have created monoclonal antibodies using these peptides for more precise detection of LT-A. Monoclonal antibodies produced from two of the peptides (numbers 33 and 36) exhibited reasonable affinity for the LT-A recombinant antigen. According to peptide prediction analysis, the sites of the three different types of peptides were also identical to those displaying true antigenicity. Additionally, it was established that the peptide predicted only by ETEC-LT-A-33 had an amino acid sequence that in fact demonstrated antigenicity. The specificity of the antibody for ETEC-LT-A-33 was validated using bacterial cells, and the impact of the antibody was determined by assessing cytokine release in infected HCT-8 cells. Antibody validation data through *in vivo* are planned for additional experiments for follow-up thesis.

## Conclusion

The goal of this study was to discover optimal candidates for the development of a vaccine or a treatment for infection with *Escherichia coli*, a major cause of diarrhea. Efficacy verification experiments were conducted using epitope peptides. The monoclonal antibodies produced in this study are useful tools for vaccine production against ETEC and helped identify peptide antigen candidates.

## Data availability statement

The original contributions presented in the study are publicly available. This data can be found here: https://figshare.com/s/84d61decb6ccd7b4d0b2.

## Ethics statement

Animal studies were reviewed and approved by the Center for Infectious Disease Research, National Institute of Health, Korea, and the Korea Centers for Disease Control and Prevention.

## Author contributions

Conceptualization, S-HC and J-YP. Writing—original draft preparation, S-HC and J-YP. Writing—review and editing, J-YP. Visualization, J-YP. Supervision, S-HC. Project administration, S-HC. Funding acquisition, S-HC. All authors contributed to the article and approved the submitted version.
